# Testing and Masking Policies and Hospital-Onset Respiratory Viral Infections

**DOI:** 10.1001/jamanetworkopen.2024.48063

**Published:** 2024-11-27

**Authors:** Theodore R. Pak, Tom Chen, Sanjat Kanjilal, Caroline S. McKenna, Chanu Rhee, Michael Klompas

**Affiliations:** 1 Department of Population Medicine, Harvard Medical School and Harvard Pilgrim Health Care Institute, Boston, Massachusetts; 2 Department of Medicine, Massachusetts General Hospital, Boston; 3 Department of Medicine, Brigham and Women's Hospital, Boston, Massachusetts; 4 Department of Population Medicine, Harvard Pilgrim Health Care Institute, Boston, Massachusetts

## Abstract

This cohort study examines the ratio between hospital- and community-onset respiratory viral infections at different levels of testing and masking from 2020 to 2024.

## Introduction

Most hospitals have stopped testing all patients for SARS-CoV-2 upon admission and requiring masking. Ten hospitals in the Mass General Brigham hospital system ended both these precautions simultaneously in May 2023 but restarted masking for health care workers in January 2024 during a winter respiratory viral surge. We characterized the association of these changes with the relative incidence of hospital-onset SARS-CoV-2, influenza, and respiratory syncytial virus (RSV).

## Methods

This cohort study was approved with a waiver of informed consent by the Mass General Brigham institutional review board and followed the Strengthening the Reporting of Observational Studies in Epidemiology (STROBE) reporting guideline. We analyzed all patients admitted between November 6, 2020, and March 21, 2024, to 10 hospitals (2 tertiary hospitals, 7 community hospitals, 1 eye and ear hospital) using a Poisson interrupted time-series design. We identified hospital-onset infections (first positive polymerase chain reaction [PCR] test more than 4 days after admission) and community-onset infections (first positive within 4 days) for SARS-CoV-2, influenza, and RSV. The study had 4 periods: pre-Omicron with universal testing and masking; Omicron with universal testing and masking; Omicron without universal testing and masking; and Omicron after restarting masking for health care workers alone. Periods with universal testing included both admission testing and serial retesting of patients who were SARS-CoV-2-negative (eMethods in Supplement 1). Adherence to testing policy was assessed using systemwide testing data. We modeled level and trend changes in the rate of hospital-onset infections relative to community-onset infections across these periods and adjusted for seasonality and seasonality-period interactions, selecting a reduced best-fit model using the Akaike information criterion (eMethods in Supplement 1).^1^ We calculated adjusted risk ratios and bootstrapped 95% CIs and then assessed statistical significance by 95% CIs that excluded 1.

We reviewed 100 randomly selected hospital-onset SARS-CoV-2 cases admitted after universal testing ended, to assess whether community-onset cases were being misclassified as hospital-onset using 3 yes or no characteristics: new symptoms of respiratory infection, known exposure to SARS-CoV-2, and PCR cycle threshold of less than 30. All analyses were performed in R version 4.2.1 (R Project for Statistical Computing). Data were analyzed from December 19, 2023, to October 7, 2024.

## Results

Among 641 483 admissions (357 263 women [55.7%]; median [IQR] age, 61 [38-74] years), there were 30 071 community-onset and 2075 hospital-onset SARS-CoV-2, influenza, and RSV infections (Table). While universal testing was in effect, admission SARS-CoV-2 tests were collected for 386 257 of 415 541 admissions (92.9%), compared with 39 765 of 149 712 admissions (26.5%) after stopping universal testing. The median (IQR) interval between tests in admissions of 8 days or more was 4.4 (3.4-6.1) days during universal testing vs 11.1 days (8.4-17.0) days after stopping universal testing.

**Table.** Changes in Hospital-Onset vs Community-Onset Respiratory Viral Infections After Omicron Dominance, Ending Universal Masking and SARS-CoV-2 Testing, and Restarting Masking of Health Care Workers

Characteristic	Patients, No. (%)			
	Pre-Omicron	Omicron		
	Universal masking and testing (n = 206 911)^a^	Universal masking and testing (n = 264 338)^b^	After universal masking and testing ended (n = 127 305)^c^	After restarting masking of only staff (n = 42 929)^d^
**Hospital-onset cases**				
Cases, No.	275	1119	500	181
Infection type				
SARS-CoV-2	267 (97.1)	1054 (94.2)	436 (87.2)	107 (59.1)
Influenza	1 (0.4)	53 (4.7)	35 (7.0)	58 (32.0)
RSV	7 (2.5)	24 (2.1)	33 (6.6)	25 (13.8)
Sex^e^				
Female	111 (40.4)	504 (45.0)	233 (46.6)	78 (43.1)
Male	164 (59.6)	612 (54.7)	264 (52.8)	103 (56.9)
Age, median (IQR), y	62 (46 to 75)	65 (42 to 79)	70 (50 to 81)	68 (49 to 79)
Time to first positive test, median (IQR), d	7.9 (5.1 to 15.1)	9.6 (5.8 to 21.7)	10.0 (6.2 to 18.8)	8.0 (5.8 to 19.2)
**Community-onset cases**				
No. of cases	8868	14 998	3996	2209
Infection type				
SARS-CoV-2	8446 (95.2)	12 482 (83.2)	2941 (73.6)	1066 (48.3)
Influenza	83 (0.9)	1480 (9.9)	454 (11.4)	864 (39.1)
RSV	345 (3.9)	1116 (7.4)	629 (15.7)	313 (14.2)
Sex^e^				
Female	4200 (47.4)	7874 (52.5)	2124 (53.2)	1171 (53.0)
Male	4628 (52.2)	7072 (47.2)	1865 (46.7)	1031 (46.7)
Age, median (IQR), y	65 (52 to 77)	68 (55 to 78)	67 (54 to 77)	65 (57 to 76)
Time to first positive test, median (IQR), d	0.1 (−0.8 to 0.2)	0.1 (0.1 to 0.2)	0.1 (0 to 0.2)	0.1 (0.1 to 0.2)
**Changes between study periods**				
Weekly hospital-onset to community-onset case ratio, mean (95% CI), %^f^	2.9 (NA)	7.6 (6.0 to 9.1)	15.5 (13.6 to 17.4)	8.0 (5.0 to 11.0)
Adjusted rate ratio for hospital-onset infections vs the counterfactual of no change from the prior period, No. (95% CI)^f,g^	1 [Reference]	2.62 (1.89 to 3.56)	1.25 (1.02 to 1.53)	0.67 (0.52 to 0.85)
*P* value	NA	<.001	.03	.001

Abbreviations: NA, not applicable; RSV, respiratory syncytial virus.

^a^ From November 6, 2020, to December 16, 2021.

^b^ From December 17, 2021, to May 11, 2023.

^c^ From May 12, 2023, to January 1, 2024.

^d^ From January 2, 2024, to March 21, 2024.

^e^ Sex assigned at birth.

^f^ Includes infections by SARS-CoV-2, influenza, and RSV.

^g^ Calculated using Poisson regression, with adjustment for community-onset infections, seasonality, and seasonality-period interactions, followed by stepwise model selection by Akaike information criterion (Supplement 1).

In unadjusted analyses, the mean weekly ratio between hospital-onset and community-onset infections increased from 2.9% prior to Omicron dominance to 7.6% (95% CI, 6.0%-9.1%) during Omicron dominance. After universal masking and testing ended, it increased to 15.5% (95% CI, 13.6%-17.4%), then fell to 8.0% (95% CI, 5.0%-11.0%) following resumption of masking among health care workers. Under the adjusted Poisson model (Figure), cessation of universal masking and testing was associated with a 25% increase in hospital-onset respiratory viral infections compared with the preceding Omicron-dominant period (rate ratio [RR], 1.25; 95% CI, 1.02-1.53), and resumption of masking among staff was associated with a 33% decrease in hospital-onset respiratory viral infections (RR, 0.67; 95% CI, 0.52-0.85). Among 100 randomly selected hospital-onset SARS-CoV-2 cases detected after universal testing ended, 89 (89.0%) had new symptoms, 27 (27.0%) had known SARS-CoV-2 exposures, 80 (80.0%) had PCR cycle thresholds of 30 or less, 97 (97.0%) met 1 or more of the preceding criteria, and 8 (8.0%) died in-hospital.

**Figure.**
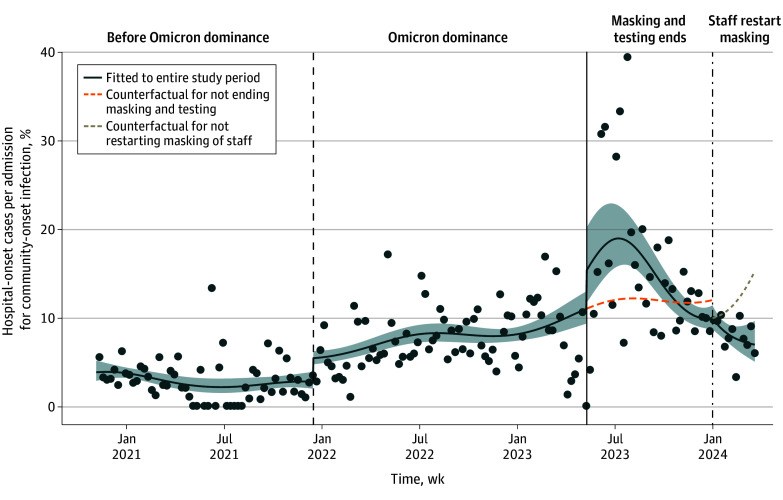
Weekly New Hospital-Onset Cases of SARS-CoV-2, Influenza, and RSV Infection Per Admission for Community-Onset Infection at 10 US Hospitals Mean weekly rate ratios of new hospital-onset infections vs new admissions for community-onset infection by SARS-CoV-2, influenza, and respiratory syncytial virus (RSV) (dots). Hospital-onset infections were defined as a diagnosis more than 4 days after arrival, and community-onset infections diagnosed 4 days or less from arrival. The dashed vertical line denotes when Omicron became the dominant variant in Massachusetts (more than 50% of sequenced samples). The solid vertical line demarcates when universal admission testing and masking ended. The dot-dashed vertical line denotes when masking of only health care workers was restarted. The dark blue solid line indicates the Poisson regression model fit to the entire study period, and the shaded area represents a 95% CI. The model under the counterfactual scenario of not ending universal admission testing and masking is drawn as a dashed orange line, and the counterfactual for not restarting masking of staff is drawn as the dashed light brown line.

## Discussion

In this study, stopping universal masking and SARS-CoV-2 testing was associated with a significant increase in hospital-onset respiratory viral infections relative to community infections. Restarting the masking of health care workers was associated with a significant decrease. Limitations of our analysis included a lack of concurrent controls, possible variations in compliance, difficulty disentangling effects of testing vs masking, and potential case misclassification. However, medical record reviews suggested most hospital-onset cases were true acute cases.

Nosocomial respiratory viral infections remain associated with increased length of stay and higher mortality in hospitalized populations.^2,3,4^ Our data suggest that masking^5^ and testing^6^ were 2 potentially effective measures to protect patients who are hospitalized, particularly when community respiratory virus incidence rates were elevated.

## Notes

Supplement 1.
eMethods.

Supplement 2.
Data Sharing Statement
