# Supplementary material for: Testing and Masking Policies and Hospital-Onset Respiratory Viral Infections
Source: JAMA Netw Open. 2024 Nov 27;7(11):e2448063. doi: 10.1001/jamanetworkopen.2024.48063 (PMC12549082; doi:10.1001/jamanetworkopen.2024.48063)
Supplement: Supplement 1. — eMethods. [file jamanetwopen-e2448063-s001.pdf]

Supplemental Online Content

Pak T, Chen T, Kanjilal S, McKenna CS, Rhee C, Klompas M. Testing and masking policies and hospital-onset respiratory viral infections. *JAMA Netw Open*. 2024;7(11):e2448063. doi:10.1001/jamanetworkopen.2024.48063

eMethods.

This supplemental material has been provided by the authors to give readers additional information about their work.

© 2024 Pak T et al. *JAMA Network Open.*

eMethods

Masking Policies

From November 6, 2020 (the start date of the study) through May 11, 2023, all Mass General Brigham (MGB) staff, patients, and visitors were required to wear hospital-issued surgical or procedural masks during all interactions. N95 respirators (along with eye protection, gown, and gloves) were required when caring for patients with suspected or confirmed COVID-19.

From May 12, 2023 to January 1, 2024, all staff, patients, and visitors were only asked to wear a mask if they had current symptoms of a respiratory illness, if they had a known COVID-19 infection and were within the isolation period, or if they had a known recent exposure to COVID-19. Requirements for staff to mask while caring for patients with suspected or confirmed respiratory viral infections were unchanged.

From January 2, 2024 to March 21, 2024, all staff were again required to wear a hospital-issued surgical or procedural mask during direct interactions with patients in clinical care locations (and not in other locations, such as hallways, elevators, or waiting areas). Patients and visitors were encouraged but not required to wear a mask in clinical care locations.

Testing Policies

From November 6, 2020 (the start date of the study) through May 11, 2023, all patients were required to undergo SARS-CoV-2 testing via PCR of a nasal or nasopharyngeal swab on admission plus a second test 72h after admission. In early 2022, automated serial SARS-CoV-2 testing every 5 days starting after the second negative admission test was also implemented. Testing was also performed for any new symptoms concerning for COVID-19 and within 72h of aerosol-generating procedures.

After May 12, 2023, all automated SARS-CoV-2 testing of asymptomatic patients ceased. Testing was instead performed only for patients with new symptoms concerning for COVID-19 or another valid clinical indication.

We assessed adherence to the admission testing policy using laboratory information system data from MGB's microbiology laboratory. The presence of a SARS-CoV-2 PCR test collected up to 7 days before and up to 3 days after the time of the patient's arrival to the hospital counted as a valid admission test. Hospital admissions shorter than 24h were excluded when assessing testing policy adherence. The interval between serial tests was calculated by excluding admissions with positive viral respiratory tests and averaging the amount of time between each SARS-CoV-2 test and the subsequent test or discharge event per admission. The distributions of these per-admission averages before and after the end of the universal testing policy (May 11, 2023) were summarized using the median and interquartile range.

Unadjusted Analyses

For the unadjusted comparisons of the mean weekly ratio between hospital-onset and community-onset respiratory viral infections between study periods, we used a piecewise linear model where the outcome variable was this weekly ratio, and the dependent variables were dummy variables representing each transition between the four study periods (pre-Omicron with universal testing and masking; Omicron with universal testing and masking; Omicron after stopping universal testing and masking; and Omicron after restarting masking of healthcare workers). We used the model coefficients to estimate changes in the mean weekly ratio between study periods and calculated 95% confidence intervals using the standard errors of these coefficients.

Poisson Model Details

Let $t_1, \cdots, t_n$ represent the timepoints of data collection, measured in days from the start of the study period. We

© 2024 Pak T et al. *JAMA Network Open.*

model $\mu(t)$, the expected number of hospital-onset infections in the week preceding timepoint t, as follows:

$$\begin{aligned}\log(\mu(t)) = \log(X(t)) &+ \left(\alpha_0 + \alpha_1 t + \alpha_2 \sin(y(t)) + \alpha_3 \cos(y(t))\right) \\ &+ B_t\left(\beta_0 + \beta_1 (t-\tau_1)^+ + \beta_2 \sin(y(t)) + \beta_3 \cos(y(t))\right) \\ &+ C_t\left(\gamma_0 + \gamma_1 (t-\tau_2)^+ + \gamma_2 \sin(y(t)) + \gamma_3 \cos(y(t))\right) \\ &+ D_t\left(\delta_0 + \delta_1 (t-\tau_3)^+ + \delta_2 \sin(y(t)) + \delta_3 \cos(y(t))\right)\end{aligned}$$

where $X(t)$ is the count of community-onset infections in the week preceding timepoint t; $y(t) = 2\pi t/365$ is the scaled time to account for seasonality with a periodicity of 365 days; and $\alpha, \beta, \gamma, \delta$ are coefficients associated with each of the four study periods, namely:

- $\alpha \rightarrow$ pre-Omicron with universal testing and masking
- $\beta \rightarrow$ Omicron with universal testing and masking, starting on day τ_1 (December 17, 2021)
- $\gamma \rightarrow$ Omicron after stopping universal testing and masking, starting on day τ_2 (May 12, 2023)
- $\delta \rightarrow$ Omicron after restarting masking of healthcare workers, starting on day τ_3 (January 2, 2024)

Specifically, α_0 describes the overall level change starting in the pre-Omicron period, α_1 describes trend change, (α_2, α_3) are terms for Fourier seasonality adjustment, and likewise for (β, γ, δ) and their respective periods. Furthermore, $B_t = 1$ for all $t > \tau_1$ and is 0 otherwise; $C_t = 1$ for all timepoints $t > \tau_2$ and is 0 otherwise; and $D_t = 1$ for all timepoints $t > \tau_3$ and is 0 otherwise.

Now, let $(\hat{\mu}, \hat{\alpha}, \hat{\beta}, \hat{\gamma}, \hat{\delta})$ be estimates of these parameters obtained from fitting the model to the data, with backward stepwise elimination of parameters using the Akaike Information Criterion (AIC). The estimated total number of hospital-onset cases over timepoints $t_1, \cdots, t_n$ (spaced seven days apart) is then

$$\hat{N}_{HO} = \sum_{i=1}^{n} \hat{\mu}(t_i)$$

An averaged rate ratio over an entire period (e.g., Omicron after stopping admission testing and masking, containing timepoints $s_1, \cdots, s_m$) between the predicted risk and the counterfactual of no changes from the prior period would be estimated as

$$\widehat{RR}_{HO} = \frac{\sum_{j=1}^{m} \hat{\mu}(s_j | \gamma = \hat{\gamma})}{\sum_{j=1}^{m} \hat{\mu}(s_j | \gamma = 0)}$$

This approach retains adjustments for both the community-onset infection rate and seasonality. For 95% confidence intervals, we use 10,000 bootstrap samples from the multivariate normal distributions implied by the variance-covariance matrix of the fitted model and extract the 0.025 and 0.975 quantiles from the corresponding distribution of $\widehat{RR}_{HO}$. To calculate the p-value from these bootstrap samples, we compute the proportion of these bootstrapped estimates that are greater than one and the proportion that are less than one. The p-value is calculated by taking the smaller of these two proportions and doubling it, representing the likelihood of observing the effect size we found or more extreme, assuming there is no true effect (i.e., a risk ratio of one). All analyses were performed in R version 4.2.1.

© 2024 Pak T et al. *JAMA Network Open.*
