# Supplementary material for: Testing and Masking Policies and Hospital-Onset Respiratory Viral Infections
Source: JAMA Netw Open. 2024 Nov 27;7(11):e2448063. doi: 10.1001/jamanetworkopen.2024.48063 (PMC12549082; doi:10.1001/jamanetworkopen.2024.48063)
Supplement: Supplement 2. — Data Sharing Statement [file jamanetwopen-e2448063-s002.pdf]

Data Sharing Statement

Data
Data available: No

Additional Information
Explanation for why data not available: Individual patient-level data cannot be made available because the analysis requires the use of real admission and diagnosis dates, which are considered identifiers under the HIPAA Safe Harbor rule, i.e., the dataset contains protected health information. However, on request, we can share the R code created for the statistical analysis.
